# A glycolysis-related gene expression signature in predicting recurrence of breast cancer

**DOI:** 10.18632/aging.103806

**Published:** 2020-11-16

**Authors:** Jianing Tang, Yongwen Luo, Gaosong Wu

**Affiliations:** 1 Department of Thyroid and Breast Surgery, Zhongnan Hospital of Wuhan University, Wuhan, China; 2 Department of Urology, Zhongnan Hospital of Wuhan University, Wuhan, China

**Keywords:** breast cancer, glycolysis, prognosis

## Abstract

Metabolic change is the hallmark of cancer. In the present study, we aimed to develop a glycolysis-related gene signature to predict the prognosis of breast cancer patients. Gene expression profiles and clinical data of breast cancer patients were obtained from the GEO database. A four-gene based signature (ALDH2, PRKACB, STMN1 and ZNF292) was developed to separate patients into high-risk and low-risk groups. Patients in the low-risk group had significantly better prognosis than those in the high-risk group. Time-dependent ROC analysis demonstrated that the glycolysis-related gene signature had excellent prognostic accuracy. We further confirmed the expression of the four prognostic genes in breast cancer and paracancerous tissue samples using qRT-PCR analysis. Expression level of PRKACB was higher in paracancerous tissues, while STMN1 and ZNF292 were overexpressed in tumor samples, no significant difference was observed in ALDH2 expression level. Global proteome data of 105 TCGA breast cancer samples obtained from the Clinical Proteomic Tumor Analysis Consortium (CPTAC) were used to evaluate the prognostic value in protein levels. Consistently, high expression level of PRKACB protein was associated with favorable prognosis, while high ZNF292 and STMN1 protein expression levels indicated poor prognosis. The glycolysis-related gene signature might provide an effective prognostic predictor and a new insight for individualize management of breast cancer patients.

## INTRODUCTION

Breast cancer is the most common malignant tumor and is also the leading cause of cancer death among women around the world. According to the Global Cancer Statistics 2018, breast cancer accounts for 24% of the total female cancer cases and 15% of the total female cancer mortality [1]. Although incidence of breast cancer is markedly higher in developed countries, almost 50% of new breast cancer diagnoses and approximately 60% of breast cancer deaths occur in developing countries. Breast cancer survival rate also varies largely worldwide, 5-year survival rate is estimated at 80% in developed countries while 40% in developing countries [2]. With the improvement of therapeutic strategies, breast cancer-related deaths have decreased in recent decades. Unfortunately, some breast cancer patients initially diagnosed with advanced stage are still incurable, and nearly 30% patients diagnosed at early stage will eventually develop locoregional or distant tumor recurrence [3]. In the majority of breast cancer patients, metastatic disease is the underlying cause of death and current clinical strategies fall short in accurately identifying patients at high risk of recurrence. It is important to understand the molecular mechanisms underlying the recurrent process of breast cancer and innovative biomarkers for prognosis predication.

The metabolic alterations are hallmarks of cancer cells, which could distinguish them from the normal cells. This altered metabolism is necessary for cancer cells to sustain high proliferative rates in a hostile environment [4]. Under aerobic conditions, glucose is first processed to pyruvate in the cytosol via glycolysis, and thereafter to CO_2_ in the mitochondria. Under anaerobic conditions, most cells prefer the glycolysis, little pyruvate is dispatched to the oxygen-consuming mitochondria. In the 1920s, Otto Warburg first observed a unique metabolic phenotype of cancer cells: even in the presence of oxygen, cancer cells can reprogram their glucose metabolism, thus enhance glycolysis and reduce oxidative phosphorylation, which has been termed ‘‘aerobic glycolysis” [5, 6]. Upregulated glycolysis is associated with the capabilities of attenuation of apoptosis, avoidance of cytostatic controls, and cell proliferation [7]. Activation of oncogenes and mutations of tumor suppressors are implicated in the metabolism of breast cancer cells. The MYC proto-oncogene is a critical regulator of cell proliferation, differentiation, and apoptosis [8]. In addition, c-MYC activation upregulates expression of glycolytic target genes (PFK1, GLUT, ENO, and HK) and LDH, which contribute directly to the aerobic glycolysis [9]. Under hypoxic conditions, HIF1α upregulates glycolysis and downregulates phosphorylation by inducing expression of glucose transporters, glycolytic enzymes, pyruvate dehydrogenase kinase 1 and lactate dehydrogenase A [10–12]. Wild type TP53 inhibits the expression of glucose transporters and glycolytic enzymes, thereby suppressing glycolysis [13, 14]. Targeting glycolysis is a promising therapeutic strategy for cancer therapy.

In the present study, we identified a glycolysis-related risk signature and constructed a nomogram for patients with breast cancer. These results might provide an effective prognostic predictor and a new view for individual treatment of breast cancer patients.

## RESULTS

### Patient characteristics

**Figure 1:**
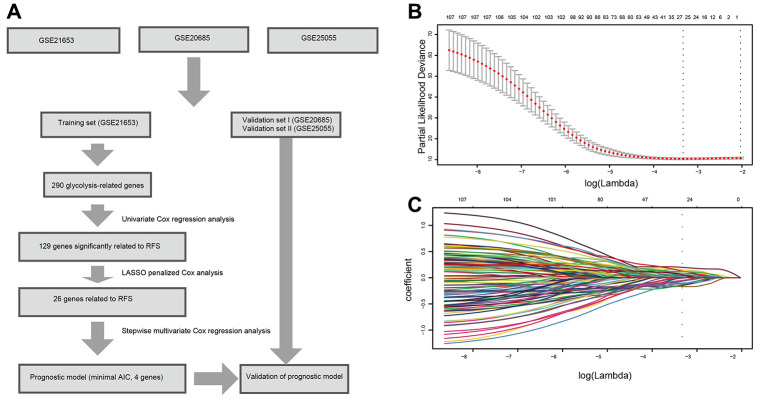
**Flow chart and 10-time cross-validation for tuning parameter selection.** (**A**). Flow chart indicating the process of selecting target genes in this study. (**B**) Ten-time cross validation for tuning parameter selection in the lasso model. (**C**). LASSO coefficient profiles of the 129 prognostic genes. A vertical line is drawn at the value chosen by 10-fold cross-validation. *Abbreviations: AIC (Akaike information criterion), RFS (relapse-free survival).*

A flow chart of this study was shown in Figure 1. A total of 878 patients from three datasets (GSE21653, GSE20685, and GSE25055) were included in our analysis. 241 patients from GSE21653 were assigned as the training set, 327 patients from GSE20685 and 310 patients from GSE25055 were assigned in the validation sets. In GSE21653, median follow-up time was 5.73 years in the low-risk group and 2.99 years in the high-risk group. 28 patients in the low-risk group (20.1%) and 48 patients in the high-risk group (47.1%) had locoregional or distant recurrence during the follow-up period. Similar results were found in the validation sets (Table 1).

**Table 1:** Clinicopathologic characteristics of three sets of breast cancer patients according to the integrated mRNA signature.

**Variables**	**GSE21653 (n=241)**		**GSE20685 (n=327)**		**GSE25055 (n=310)**	
	**Low risk (%)**	**High risk (%)**	**Low risk (%)**	**High risk (%)**	**Low risk (%)**	**High risk (%)**
Age at diagnosis (years)						
Median	55	56	47	44	48	50
≤ 50	44(31.7)	38(37.3)	120(63.5)	89(64.5)	100(55.2)	68(52.7)
> 50	95(68.3)	64(62.7)	69(36.5)	49(35.5)	81(44.8)	61(47.3)
T stage						
T0	0	0	0	0	0	2(1.6)
T1	32(23.0)	23(22.5)	68(36.0)	33(23.9)	16(8.8)	4(3.1)
T2	73(52.5)	48(47.1)	108(57.1)	80(58.0)	101(55.8)	64(49.6)
T3	34(24.5)	31(30.4)	9(4.8)	17(12.3)	39(21.5)	35(27.1)
T4	0	0	4(2.1)	8(5.8)	25(13.8)	24(18.6)
Lymph node status						
Negative	62(44.6)	51(50.0)	94(49.7)	43(31.2)	67(37.0)	20(15.5)
Positive	77(55.4)	51(50.0)	95(50.3)	95(68.8)	114(63.0)	109(84.5)
Metastases status						
Negative			188(58.9)	1(12.5)		
Positive			131(41.1)	7(87.5)		
Grade						
I	37(26.6)	6(5.9)			16(8.8)	3(2.3)
II	60(43.2)	23(22.5)			90(49.7)	27(20.9)
III	42(30.2)	73(71.6)			65(35.9)	86(66.7)
Indeterminate					10(5.5)	13(10.1)
Molecular subtype						
Basal	20(14.4)	53(52.0)			29(16.0)	93(72.1)
ERBB2	11(7.9)	10(9.8)			13(7.2)	7(5.4)
Luminal	90(64.7)	33(32.3)			109(65.7)	24(18.6)
Normal like	18(12.9)	6(5.9)			20(11.0)	5(3.9)
Disease-relapse event	28(20.1)	48(47.1)	75(30.7)	63(75.9)	14(7.7)	52(40.3)
Median Follow-up (years)	5.73	2.99	8.7	5.7	3.0	1.8

### Establishment of the prognostic glycolysis-related gene signature

**Figure 2:**
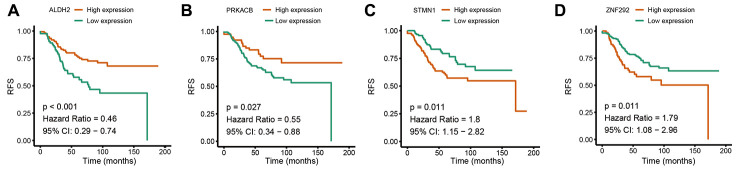
**Univariate Cox regression analysis of the four prognostic genes in the signature.** (**A**) ALDH2. (**B**) PRKACB. (**C**) STMN1. (**D**) ZNF292. *Abbreviations: RFS (relapse-free survival).*

**Figure 3:**
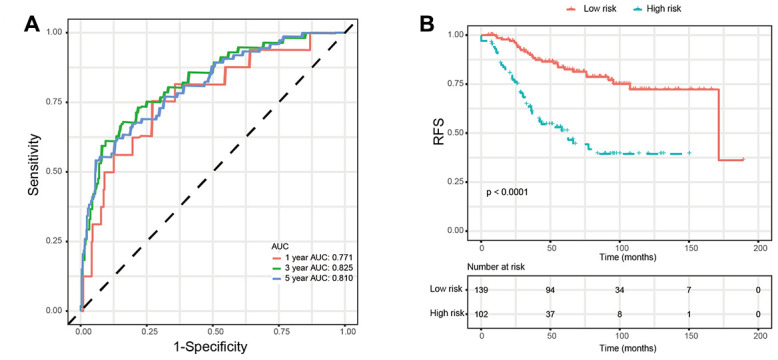
**Validation of prognostic risk score model in training set.** (**A**) Time-dependent ROC curves of the glycolysis-related signature. (**B**) Kaplan-Meier survival analysis of the glycolysis-related signature. *Abbreviations: RFS (relapse-free survival).*

**Figure 4:**
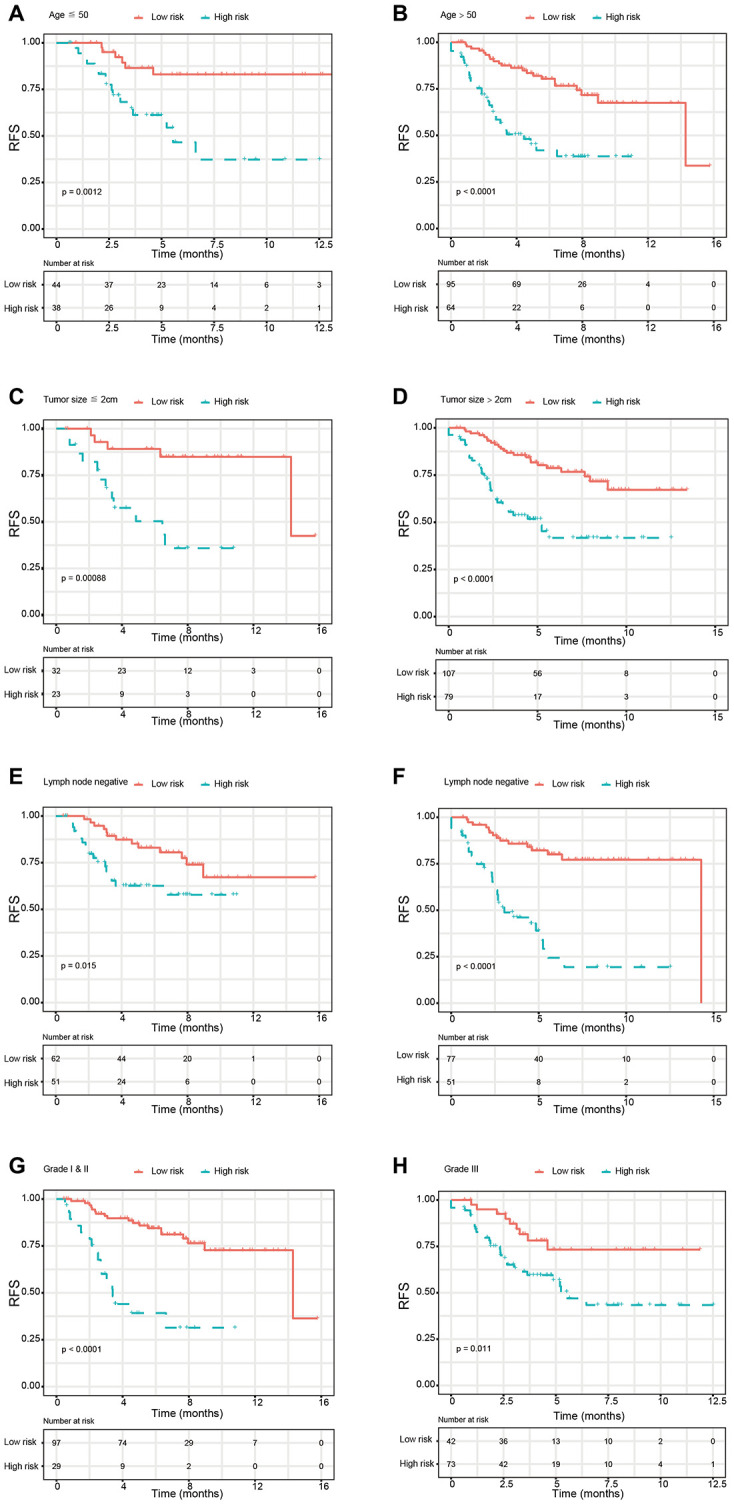
**Kaplan-Meier survival analysis for patients according to the glycolysis-related signature stratified by clinicopathological risk factors.** (**A**, **B**) Age. (**C**, **D**) Tumor size. (**E**, **F**) Lymph node status. (**G**, **H**). Tumor grade. *Abbreviations: RFS (relapse-free survival).*

**Figure 5:**
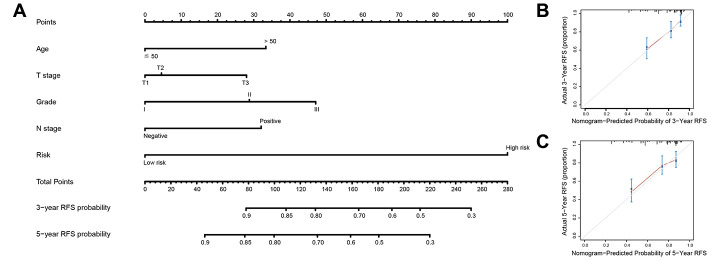
**Nomogram to predict risk of cancer recurrence.** (**A**) Nomograms to predict risk of cancer recurrence. (**B**) 3-year nomogram calibration curves in training set. (**C**) 5-year nomogram calibration curves in training set. *Abbreviations: RFS (relapse-free survival)*.

Based on the univariate Cox regression analysis, 129 glycolysis-related genes significantly associated with the prognosis of breast cancer patients were considered as prognostic genes and they were included in the subsequent analysis. After primary filtration, we used Lasso regression analysis to further narrow the genes for the construction of prognostic model. As a result, 26 glycolysis related genes were screened out for the stepwise multivariate Cox regression analysis. And four genes were finally selected to construct a prognostic model. Prognostic score was calculated for every patient based on the expression levels of the four genes and weighted by the multivariate Cox regression coefficients as follows: prognostic score = (-0.34 × expression level of ALDH2) + (-0.28 × expression level of PRKACB) + (0.33 × expression level of ZNF292) + (0.32 × expression level of STMN1). Consistent with the univariate Cox regression analysis, ALDH2 and PRKACA showed negative coefficients in the prognostic model, indicating their expression was associated with better relapse free survival (RFS). ZNF292 and STMN1 had positive coefficients, their high expression implying a shorter RFS (Figure 2). Patients in training set were divided into low-risk group and high-risk group based on the optimal cut-off value of prognostic score (0.05). As shown in Figure 3, time-dependent ROC analysis demonstrated that this signature had good prognostic accuracy. AUCs of the four-gene prognostic model were 0.771, 0.825 and 0.810 at 1, 3, and 5-year RFS time. Kaplan-Meier survival analysis indicated patients in the high-risk group had significantly poor RFS rate. To confirm the prognostic value of the glycolysis-related signature in different population, we stratified patients by clinicopathological risk factors. We also found that prognosis of patients in the low-risk group was more favorable than those in the high-risk group (Figure 4). We further developed a nomogram combining the glycolysis-related signature and clinicopathological risk factors to provide a quantitative method for prediction of 3- and 5-year RFS. Calibration plots indicated that the nomogram had a similar performance compare to the ideal model (Figure 5). When adjusting for the classical clinicopathologic factors, multivariate Cox regression analysis demonstrated that the glycolysis-related gene signature was independently associated with the RFS time of breast cancer patients (Table 2).

**Table 2:** Multivariate Cox proportional hazards regression analysis of the clinicopathologic characteristics and the glycolysis-related signature with RFS.

**Variable**	**Training set**		**Validation set I**		**Validation set II**	
	**HR (95%Cl)**	**P**	**HR (95%Cl)**	**P**	**HR (95%Cl)**	**P**
**Age (> 50 vs. ≤ 50 y)**	0.706(0.479,1.041)	0.079	0.951(0.624,1.449)	0.814	0.994(0.951,1.039)	0.785
**Tumor size (≤ 2 cm vs. > 2 cm)**	1.924(1.355,2.730)	0.001	1.832(1.254,2.677)	0.002	1.267(0.959,1.674)	0.096
**Lymph node status (Negative vs. Positive)**	1.283(0.970,1.814)	0.159	1.432(0.988,2.074)	0.058	1.175(0.512,2.696)	0.703
**Tumor grade (Grade I vs. Grade II & III)**	1.271(0.986,1.640)	0.064			0.978(0.542,1.763)	0.940
**Estrogen receptor (Negative vs. Positive)**	1.475(0.935, 2.327)	0.095			0.725 (0.072, 7.250)	0.784
**Progesterone receptor (Negative vs. Positive)**	0.548 (0.224, 1.341)	0.188			0.926 (0.093, 9.238)	0.948
**HER2 (Negative vs. Positive)**	1.012 (0.671, 1.526)	0.601			1.958 (0.383, 10.010)	0.419
**Integrated RNA signature (low risk vs. high risk)**	3.453(2.412,4.943)	<0.001	4.402(2.937,6.598)	<0.001	7.902(2.846,21.941)	<0.001

### Validation of the signature

**Figure 6:**
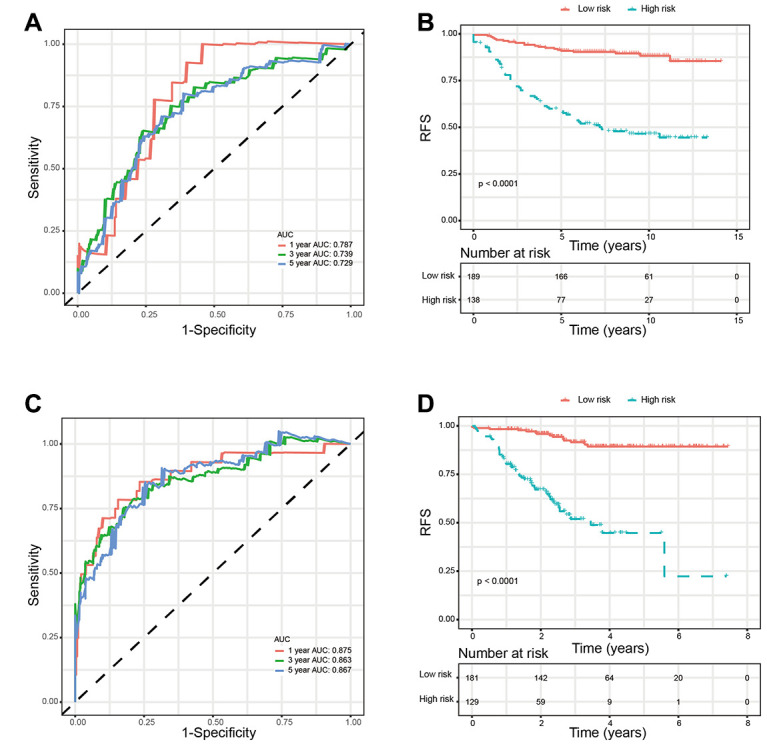
**Validation of glycolysis-related signature in validation sets.** (**A**) Time-dependent ROC curves of the glycolysis-related signature in validation set GSE20685. (**B**) Kaplan-Meier survival analysis of the glycolysis-related signature in validation set GSE20685. (**C**). Time-dependent ROC curves of the glycolysis-related signature in validation set GSE25055. (**D**). Kaplan-Meier survival analysis of the glycolysis-related signature in validation set GSE25055. *Abbreviations: RFS (relapse-free survival).*

We used two external validation sets (GSE20685 and GSE 25055) to further access the prognostic value of glycolysis-related signature. Patients in the two validation sets were separated into low-risk group and high-risk group according to the glycolysis-related signature identified above. Consistently, patients in the low-risk group showed significantly higher survival rate as compared with those in the high-risk group. In addition, the 1-year, 3-year, and 5-year AUCs demonstrated that this glycolysis-related signature had excellent prognostic accuracy (Figure 6).

### Expression validation of the four genes

**Figure 7:**
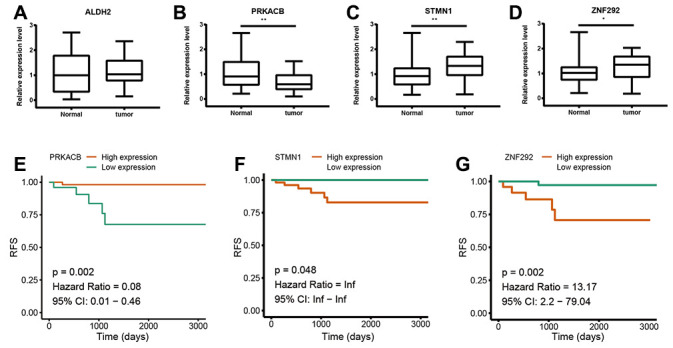
**Validation of the four prognostic genes.** (**A**–**D**). Relative mRNA expression of ALDH2, PRKACB, STMN1 and ZNF292 in breast cancer and paracancerous tissues samples. (**E**–**G**). Prognostic value of PRKACB, STMN1 and ZNF292 protein levels. *Abbreviations: RFS (relapse-free survival)*

**Figure 8:**
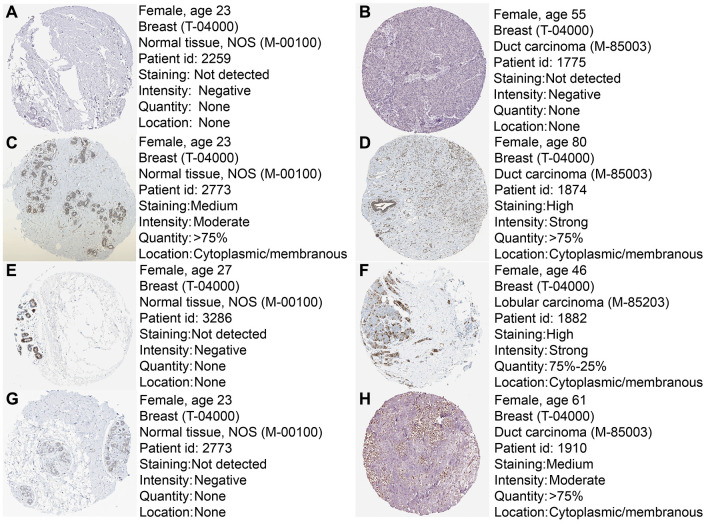
**Immunohistochemistry of the four prognostic genes based on the Human Protein Atlas.** (**A**) IHC staining of ALDH2 in normal tissue. (**B**) IHC staining of ALDH2 in tumor tissue. (**C**) IHC staining of PRKACB in normal tissue. (**D**) IHC staining of PRKACB in tumor tissue. (**E**) IHC staining of STMN1 in normal tissue. (**F**) IHC staining of STMN1 in tumor tissue. (**G**) IHC staining of ZNF292 in normal tissue. (**H**) IHC staining of ZNF292 in tumor tissue.

To further confirm the expression of the four prognostic genes, we collected breast cancer and paracancerous samples from patients undergoing modified radical mastectomy and performed qRT-PCR analysis. As show in Figure 7, expression level of PRKACB was higher in paracancerous samples, while STMN1 and ZNF292 were overexpressed in tumor samples. No difference was found in ALDH2 expression. We then investigated the prognostic value of these four genes in protein levels. High expression of PRKACB protein was associated with better RFS, while high ZNF292 and STMN1 protein expression levels indicated poor prognosis, expression of ALDH2 protein was not associated with the RFS of breast cancer patients (Figure 7E–7G). Immunohistochemistry data from the Human Protein Atlas were also used to validate the expression of prognostic genes. Consistent with our qRT-PCR results, ZNF292 and STMN1 staining were higher in tumor samples, while staining of PRKACB was lower in tumor. No difference was found in ALDH2 staining (Figure 8).

## DISCUSSION

It is well known that high rate of energy metabolism is the hallmark of cancer. In order to fuel cell growth and division, glucose uptake and utilization is markedly increased in various human cancer types. Interestingly, even in the presence of enough oxygen, malignant tumors prefer glycolysis rather than oxidative phosphorylation in the mitochondria [6]. The high rate of glycolysis not only ensures an adequate amount of energy for cancer cells, but also provide ample intermediate macromolecules to sustain a rapid cell proliferation and tumor mass expansion [15]. Thus, targeting tumor metabolism is a promising therapeutic strategy for cancer treatment. However, limited studies have systematically investigated the metabolic process of tumor and its prognostic values. And most prognostic biomarkers are identified in a wide range. Here, we investigate the associations between tumor and glycolysis to further understand the mechanism of tumorigenesis.

In the present study, we used high-throughput expression data downloaded from GEO database and constructed a glycolysis-related signature for predicting the prognosis of patients with breast cancer. After univariate, Lasso and multivariate Cox analysis, four genes (ALDH2, PRKACB, STMN1, and ZNF292) were screened out as prognostic genes for the construction of prognostic model. We first examined the prognostic value of this signature in the training set. Patients were divided into low-risk and high-risk group based on the optimal cut-off value of risk score. Kaplan-Meier survival analysis with log-rank test demonstrated that patients in low-risk group had significantly better RFS than those in the high-risk group. When stratified by clinicopathological risk factor, this glycolysis-related signature was still a significant prognostic model. The prognostic accuracy of this prognostic signature was assessed with time-dependent ROC analysis at various RFS times, AUCs of the four-gene prognostic model were 0.771 at 1 year, 0.825 at 3 years and 0.810 at 5 years. A nomogram combining the glycolysis-related signature and clinicopathological risk factors was developed to provide a quantitative method for prediction of 3- and 5-year RFS. The calibration plots demonstrated that actual survival corresponded closely with predicted survival, suggesting good predictive performance of the nomogram. Two external validation sets were used to further estimate the prognostic value of the signature. Our results indicated that this glycolysis-based classifier was still a clinically and statistically significant prognostic model. The expression levels of the four prognostic genes were further validated using breast cancer and paracancerous samples from patients undergoing modified radical mastectomy. Our qRT-PCR analysis demonstrated that STMN1 and ZNF292 were overexpressed in tumor samples, while PRKACB expression was higher in paracancerous samples. Due to the small size of the patients, no difference was found in ALDH2 expression. The same results were observed in the IHC data from the human protein atlas. Global proteome data of 105 TCGA breast cancer samples obtained from the Clinical Proteomic Tumor Analysis Consortium were used to evaluate the prognostic value of their protein levels. Consistently, high expression of PRKACB protein level was associated with better RFS, while high ZNF292 and STMN1 protein expression levels indicated poor prognosis.

The biological functions of genes in our panel have been roughly illustrated in previous studies. ALDH2 encodes a protein belonging to the aldehyde dehydrogenase family which is the second enzyme of the major oxidative pathway of alcohol metabolism. The major role of ALDH2 is to detoxify acetaldehyde (ACE) to non-toxic acetic acid. Accumulating evidences indicate that dysfunction of ALDH2 may contribute to human cancer [16, 17]. ALDH2 is suppressed in human lung adenocarcinoma, its repression leads to ACE accumulation in lung adenocarcinoma cells and induces DNA damage and metastatic features. ALDH2 suppression also promotes proliferation and stemness of lung adenocarcinoma cells both in vitro and in vivo. When Lung adenocarcinoma cells treated with ALDH2 agonist, they have suppressed proliferation, stemness and migration features [18]. In hepatocellular carcinoma, ALDH2 expression is significantly lower in tumor tissues, especially in tumors exhibited enhanced migratory capacity. Molecular biology experiments indicate that ALDH2 inhibits tumor progression largely by modulating the activity of the ALDH2-acetaldehyde-redox-AMPK axis. Thereby activating ALDH2 might be a potential strategy for the treatment of human cancers [19]. In the present study, expression of ALDH2 was negatively correlated with the RFS of breast cancer patients, while the underlying mechanisms have not been clearly investigated. The protein encoded by PRKACB is a catalytic subunit of cAMP (cyclic AMP)-dependent protein kinase. It has been demonstrated that PRKACB variants play various roles in the differentiation and proliferation of prostate cancer [20]. PRKACB is downregulated in non-small cell lung cancer (NSCLC), exogenous PRKACB inhibits the proliferation and invasion of lung cancer cells [21]. However, the molecular mechanisms for these processes remain largely unknown. Stathmin 1 (STMN1) is specifically located in cytoplasm and belongs to the Stathmin family [22]. STMN1 contributes to the microtubule catastrophe or the sequestration of alpha/beta-tubulin heterodimers. It is a critical element in regulating microtubules dynamics, which are essential for the progression of cell cycle [23, 24]. Thus, STMN1 plays a pivotal role in cell division and proliferation in cancer cells [25, 26]. The mRNA and protein level of STMN1 were significantly higher in various types of cancer, including colon cancer, lung cancer, pancreatic ductal adenocarcinoma, and hepatocellular carcinoma. In colon cancer patients, STMN1 expression is significantly related to lymph node metastasis and TNM staging. Prognosis of STMN1-positive patients is significantly poorer than STMN1-negative patients [27–30]. ZNF292 is a transcription factor-encoding gene and is considered as a candidate tumor suppressor in gastric and colorectal cancer [31]. However, high expression of ZNF292 is significantly correlated with reduced time of disease-free status in melanoma [32]. In the present study, ZNF292 expression is associated with decreased RFS of breast cancer patients. More in-depth experiments are necessary to explore the underlying mechanism of ZNF292.

In conclusion, we identified a novel glycolysis-related gene signature and constructed a nomogram that can predict the outcome of patients with breast cancer. Our results indicated this signature could be a promising prognostic target in clinical practice and provide new insights into the underlying mechanism of breast cancer.

## MATERIALS AND METHODS

### Patient clinical parameter and the gene expression data

Gene expression profiles and clinical data of breast cancer patients (GSE21653, GSE20685, and GSE25055) were downloaded from the GEO database, cases with unknown survival information were excluded from our study. GSE21653 was assigned as the training set, GSE20685 and GSE25055 were used for validation. We used the Robust Multi-array Average (RMA) method to normalize raw microarray datasets, including background correction, log2 transformation and normalization. Probes were changed into gene symbols using corresponding annotation files. We scaled the RNA expression data with a standard deviation of 1 and a mean of 0. A total of 290 glycolysis-related genes were obtained from the Molecular Signature Database v7.0.

### Construction of the prognostic gene signature and nomogram

Lasso regression analysis and Cox regression analysis were performed to identify the prognostic gene signature. Firstly, univariate Cox regression analysis was carried out to investigate the correlation between the expression level of each gene and patient survival. Genes with P value <0.05 in the univariate Cox regression analysis were considered as prognostic genes and selected for further analysis. Then we performed Lasso-penalized Cox analysis with 10-fold cross-validation to narrow the genes for prognostic prediction. 26 glycolysis-related genes were screened out. Finally, we conducted a stepwise multivariate Cox regression analysis to assess the contribution of a gene as an independent prognostic factor for patient survival. The predictive model was established by the expression levels of the glycolysis-related genes and their relative coefficient (β) derived from the multivariate Cox regression model. The prognostic score = (β × expression level of ALDH2) + (β × expression level of PRKACB) + (β × expression level of ZNF292) + (β × expression level of STMN1). Subsequently, we constructed a nomogram using “rms” R package and plotted the calibrate curve.

### Validation using proteomic data

To explore the prognostic value of the prognostic gene signature in protein levels, global proteome data of 105 TCGA breast cancer samples and corresponding clinical data were downloaded from the Clinical Proteomic Tumor Analysis Consortium (https://cptac-data-portal.georgetown.edu/cptacPublic/). The Human Protein Atlas (http://www.proteinatlas.org) was also used to validate the immunohistochemistry of prognostic genes.

### Validation in human breast cancer samples

25 paired breast cancer and paracancerous tissues samples were collected from patients undergoing modified radical mastectomy at Zhongnan Hospital of Wuhan University. All the breast tumors samples were confirmed by two pathological specialists independently. The samples were immediately frozen and stored in liquid nitrogen. We isolated total RNA from breast cancer and paracancerous tissue samples and performed qRT-PCR analysis to validate the expression of prognostic genes in human samples. This study was reviewed and approved by the Ethical Board at the Zhongnan Hospital of Wuhan University with written informed consent from all the patients.

### Statistical analysis

To investigate the prediction accuracy of the glycolysis-related classifier, we performed time-dependent receiver operating characteristic (ROC) analysis using the “survivalROC” package in R software. Patients were separated into low-risk group and high-risk groups based on the optimal cut-off value of the prognostic score calculated by ‘survminer’ package. Kaplan-Meier survival analysis was conducted to assess the survival differences between low-/high-risk groups. A two-sided log-rank test was performed using ‘survival’ package in R. Student's t test was used to compare the difference between two groups. We performed all statistical analysis using R software 3.6.1 and P < 0.05 was set as the probability value of statistical significance.

### Ethics approval

The research was carried out according to the World Medical Association Declaration of Helsinki and was approved by the Ethics Committee at Zhongnan Hospital of Wuhan University.
